# Application of Gene Network Analysis Techniques Identifies AXIN1/PDIA2 and Endoglin Haplotypes Associated with Bicuspid Aortic Valve

**DOI:** 10.1371/journal.pone.0008830

**Published:** 2010-01-21

**Authors:** Eric C. Wooten, Lakshmanan K. Iyer, Maria Claudia Montefusco, Alyson Kelley Hedgepeth, Douglas D. Payne, Navin K. Kapur, David E. Housman, Michael E. Mendelsohn, Gordon S. Huggins

**Affiliations:** 1 MCRI Center for Translational Genomics, Molecular Cardiology Research Institute, Boston, Massachusetts, United States of America; 2 Cardiothoracic Surgery Division, Tufts Medical Center, Boston, Massachusetts, United States of America; 3 Massachusetts Institute of Technology, Cambridge, Massachusetts, United States of America; Ohio State University Medical Center, United States of America

## Abstract

Bicuspid Aortic Valve (BAV) is a highly heritable congenital heart defect. The low frequency of BAV (1% of general population) limits our ability to perform genome-wide association studies. We present the application of four *a priori* SNP selection techniques, reducing the multiple-testing penalty by restricting analysis to SNPs relevant to BAV in a genome-wide SNP dataset from a cohort of 68 BAV probands and 830 control subjects. Two knowledge-based approaches, CANDID and STRING, were used to systematically identify BAV genes, and their SNPs, from the published literature, microarray expression studies and a genome scan. We additionally tested Functionally Interpolating SNPs (fitSNPs) present on the array; the fourth consisted of SNPs selected by Random Forests, a machine learning approach. These approaches reduced the multiple testing penalty by lowering the fraction of the genome probed to 0.19% of the total, while increasing the likelihood of studying SNPs within relevant BAV genes and pathways. Three loci were identified by CANDID, STRING, and fitSNPS. A haplotype within the AXIN1-PDIA2 locus (*p*-value of 2.926×10^−06^) and a haplotype within the Endoglin gene (*p*-value of 5.881×10^−04^) were found to be strongly associated with BAV. The Random Forests approach identified a SNP on chromosome 3 in association with BAV (*p*-value 5.061×10^−06^). The results presented here support an important role for genetic variants in BAV and provide support for additional studies in well-powered cohorts. Further, these studies demonstrate that leveraging existing expression and genomic data in the context of GWAS studies can identify biologically relevant genes and pathways associated with a congenital heart defect.

## Introduction

The aortic valve, as formed during embryonic heart development, is comprised of three cusps divided by three commissures. Cusp fusion, or the failure of the cusps to separate during heart development, can produce a valve with two cusps (bicuspid) or one cusp (unicuspid) [1]. In a bicuspid valve, the two conjoined cusps form a larger cusp that operates with the remaining, normal cusp to perform the valve function [2]. While the prevalence of BAV is approximately one percent of live births (13.7 per 1,000), one third of the aortic valves replaced are found to be bicuspid at the time of valve replacement [3]. Thoracic ascending aortic aneurysms (TAA) are also found in patients with BAV, sometimes even in pre-teen children.

Consistent with a strong genetic predisposition the heritability estimates of BAV range from 0.75 to 0.89 [4]. Studies conducted in 13 families with BAV suggest that BAV and TAA are independent manifestations of a single underlying gene defect with incomplete penetrance [5], [6]. NOTCH1 mutations have been found in sporadic cases of BAV [7], [8]; however, a consistent role for NOTCH1 gene variants in BAV has not been found [9]. Despite a high heritability, challenges remain in determining the genetic cause of BAV. First, few multigenerational pedigrees contain BAV as an isolated trait to enable gene identification by traditional approaches. Second, large, well phenotyped BAV cohorts are not readily available due to the relative rarity of BAV in the general population. Third, it can be difficult to determine non-invasively whether a thickened and calcified aortic valve is bicuspid or tricuspid in adults; direct, surgical inspection is often required to determine whether a BAV is present, which limits subject recruitment. Finally, the phenotypic diversity observed within BAV implies an assortment of developmental pathways ultimately converging around a single, uniquely identifiable phenotypic outcome: aortic valve formation and valve disease.

Due to the complexity of the developmental processes that contribute to heart valve formation and valve disease, candidate gene approaches are unlikely to identify the genetic determinants of BAV. The application of a broader form of analysis is needed to identify the complex genomic contributors to BAV in an unbiased manner. Traditional genome-wide association study (GWAS) approaches have identified genes that contribute to common diseases including coronary artery disease [10], [11]. GWAS studies have also proven particularly effective in identifying disease-related genes for highly heritable disorders such as age-dependent macular degeneration [12]. With the addition of haplotype-based analyses, a wide spectrum of observed variant frequencies can be useful with regard to analysis of both common, complex disorders as well as highly heritable but relatively rare disorders (such as BAV) [13], [14]. While an unbiased approach offers significant potential for discovery of single, potent, highly penetrant genetic determinants of BAV, the multiple-testing load inherent to this approach weakens our ability to detect important associations. To control for random associations, generated *p*-values typically must reach “genomic” levels [15], and adopting such stringent standards is likely at the expense of weak but biologically plausible associations, especially in settings with obligate limitations to sample sizes.

These limitations have led to the development of two primary strategies for applying gene network-based analysis to genome-wide SNP datasets. The first, a knowledge-based approach, leverages existing annotations, public expression data, previously published associated genes or chromosomal regions, and any other quantifiable information that has been the output of directed study. These data are computationally combined into interaction pathways that extend from the input genes, genomic regions, and specific protein/protein interactions into the broader pathways that these elements inhabit [16]–[18]. Thus, starting from lists of the most differentially expressed genes within a trait, the multiple gene partners, alternate transcripts, functionally similar genes, conserved structural motifs or sequences, and any other quantifiable aspect of a protein or gene region can be added, allowing for the construction of a gene network relevant to the underlying disorder in question. This final list of network genes forms a prioritized list from which available probes located within these genes (or the regulatory regions surrounding them) are selected for association analysis [19]. A related approach, employed by tools such as Endeavour [20], assumes a robustly defined set of “known genes” that make up a phenotypic signature, usually based on expression data. Endeavour applies many annotation categories and a text-based search to find genes with signatures similar to the training set, returning a rated list of genes for future study that should inform and extend the “known” training set. While this approach is quite powerful, it presupposes a well-understood and robustly described starting point in the form of a training set. The complex genetic data, much of it poorly understood or contradictory, that currently describes BAV implies the need for more inclusive initial approaches that start from the various known genes but extend from there, including while broadening what is known to also include what is likely.

The second type of analysis, a machine learning approach, leverages the ability of multiply partitioned datasets to select a subset of SNPs within the overall set of available probes most likely to be informative at defining the phenotype in question [21], [22].

Here we describe the application of these two strategies to the analysis of a genome-wide SNP dataset for BAV. Furthermore, we characterize the effects of using differing techniques within these strategies on the outputted association results. Specifically, we examine STRING[16] and CANDID [17], competing knowledge-based methods for forming genetic and protein interaction networks. We also apply two other approaches to forming an *ab initio* list of SNPs likely to have high information content: fitSNPs and Random Forests. FitSNPs are a collection of variants gleaned from exhaustive searches of public expression data and are highly likely to be functionally relevant SNPs (but are not associated with a specific disease or phenotype). Random Forests are included as an unbiased probeset, selected by perceived information content of the included SNPs relative to their capability to partition our dataset relative to trait (e.g. BAV). We hypothesize that collections of SNPs selected by multiple probe prioritization strategies within haplotype blocks strongly associated with BAV will help identify genes relevant to this disorder. The product of these studies offers a comprehensive analysis of a limited cohort while still respecting the rules of multiple testing.

## Results

### BAV Cohort

We collected DNA from 68 probands found to have BAV by direct visualization at the time of aortic valve replacement or by echocardiography. The average age of BAV Genetics Study participants at the time of enrollment was 53 years (range 18 to 85), and the majority of subjects were men (54 of 66). Echocardiography demonstrated normal aortic valve function in 18 subjects, moderate to severe aortic valve stenosis was detected in 38 subjects, and three subjects had isolated severe aortic valve regurgitation. We included six subjects that had coarctation of the aorta (all repaired) and 19 who had TAA (17 repaired) in our cohort since the BAV-associated aortic phenotypes are considered to be independent manifestations of a single underlying gene defect with incomplete penetrance (5). Genomic DNA from these individuals was genotyped on the Illumina 370CNV array. Additionally, genotypes for 823 control individuals were obtained from Illumina iControlDB [23] and joined seven BAV-negative familial controls present in our dataset for a total of 830 individuals in the control cohort. After excluding the copy number probes (which are not represented in the Illumina-supplied control population), the dataset included 311,399 probes. The prevalence of BAV in the general population would suggest that only ∼1% of the control cohort would have a BAV. There was no evidence of significant population structure in the combined control/experimental group [24].

### Sources of Biological Knowledge for Our Initial Analysis

Several sources of information served as the basis for our network analysis. First, we selected MeSH terms (including OMIM headings) that characterize BAV and its associated syndromes. Second, we identified genes differentially expressed in the aorta of subjects with BAV compared with a normal tricuspid aortic valve (TAV) from the Gene Expression Omnibus (GEO) dataset GDS2922.[25] Analysis of these arrays was carried out by both parametric (limma) and non-parametric (RankProd) methods. These two approaches predominantly selected different gene-sets relative to observed expression levels in experimental (BAV, TAA) versus control (TAV, TAA) patients. Combining the output from the parametric and non-parametric array analyses resulted in a collection of 1,552 differentially expressed genes (limma n = 903, RankProd n = 649) that broadly mirrored previous findings from the datasets in question but were necessarily more inclusive. Gene ontology analysis showed a broad representation from across the functional spectrum with specific ontologic classes, such as coagulation and inflammatory response, metabolism, development, and cell communication (see Table S1). Third, we included the most significantly altered genes (n = 41) detected in the peripheral blood of patients with TAA compared with normal individuals (GSE9106).[26] Finally, we included chromosomal loci linked with BAV by microsatellite-based study [9], [27]. Through our primary analysis of microarray data and the selection of all published and genomic loci we constructed a knowledgebase for BAV to serve as a basis for the construction of an overall BAV-related gene network.

### STRING and CANDID Generated Networks and fitSNPs

**Figure 1:**
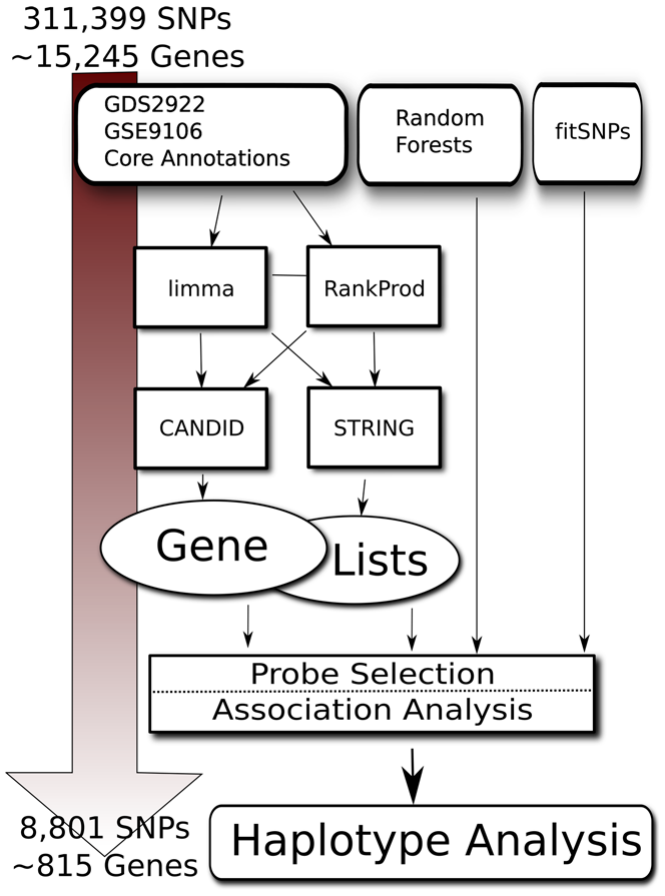
Schematic Representation of SNP Prioritization. The 311,399 SNPs present on the Illumina CNV370 (representing about 15,000 well annotated genes) are prioritized by three methods. The “knowledge-based” approach involves expression analysis and inclusion of existing annotations, term extension by CANDID or STRING network-based analysis, and ultimately leading to the output of a gene list. Probes inside and within regulatory regions of these genes are then selected for association analysis. Probe results for the STRING arm are shown as an example: 8,801 SNPs representing approximately 815 prioritized genes. In parallel, SNPs are prioritized by Random Forest analysis, which recursively partitions the data to reveal SNPs with highest likelihood of successful association results. Finally, fitSNPs (derived independently of this study) represented on the CNV370 array are analyzed for association.

We combined the multiple sources of biological knowledge, including the RankProd and limma analyses, to create parallel interaction networks relevant to BAV using STRING [16] and CANDID [17] (Figure 1). Table 1 describes the commonalities and differences of the SNPs selected by these two programs. STRING took the outputs of the limma and RankProd expression analyses and created substantially different output networks, reflecting the nature of the input, with RankProd ultimately generating a gene list composed of 6,651 prioritized SNPs, and limma 3,970 SNPs. An additional 694 probes were shared between methods. CANDID, alternatively, produced highly similar lists from the same limma and RankProd inputs, ultimately differing at only 155 out of 13,769 selected SNPs.

**Table 1:** Prioritized Probe distribution by category.

Sub-Class	CANDID	STRING
**limma only**	94	2960
**RankProd only**	61	5200
**limma/RankProd**	12,405	641
**Total SNPs**	**12560**	**8801**
**Random Forests**	222	180
**fitSNPs**	554	543
**STRING/CANDID**	1397	1397

RF and fitSNP categories in italics represent shared probes from those respective classes; STRING/CANDID row presents STRING probes in common with CANDID and vice versa.

Separately, we identified the 8,801 Functionally Interpolating SNPs (fitSNPs) present on the Illumina 370CNV array. FitSNPs were derived through extensive analysis of public array datasets [28]. While not selected relative to specific disease or expression outcome, fitSNPs represent a set of markers enriched for functionally relevant variants (e.g. non-synonymous coding region polymorphisms) from across the genome.

From the 370CNV array, 1532 probes were selected both by CANDID (12% of all CANDID SNPs) and by STRING (15% of all STRING SNPs). The fitSNPs overlapped CANDID at 554 probes (4.4%) and STRING at 543 probes (6.2%). 97 SNPs were selected by these three approaches (CANDID, STRING and fitSNPs). Full comparisons between probes selected by CANDID, STRING, Random Forests, and fitSNPs are shown in Tables 1 and 2.

**Table 2:** *ab initio* Probe distribution by category: Categories in italics represent shared probes from those respective classes.

Sub-Class	fitSNPs	Random Forests
**Random Forests**	*123*	6322
**fitSNPs**	8100	*123*
**Prioritized**	*1097*	*402*

We considered genes identified by more than one strategy to have the greatest potential for a role in BAV. To test this hypothesis we determined the association of each prioritized SNP for differentiating case (BAV) from control status. When we compared the top 100 SNPs (by *p*-value) from each strategy, three chromosomal regions were selected by all three strategies: AXIN1-PDIA2, ENG, and BAT2-BAT3.

### AXIN1-PDIA2 Haplotype Is Associated with BAV

**Figure 2:**
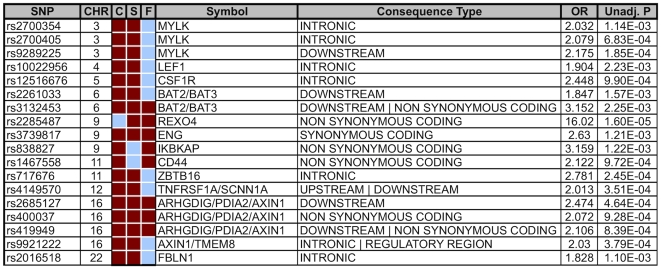
Frequent Placement in Top 100 uncorrected p-Values sorted by chromosome; SNPs appearing two or more times amongst the three categories. **C = CANDID, S = STRING, F = fitSNP**. Red = present in top 100, blue = not present in top 100. Remaining fields are Gene **symbol**, Ensembl-51 **consequence type**, **O**dds **R**atio, and unadjusted p-Value for each reported SNP.

**Figure 3:**
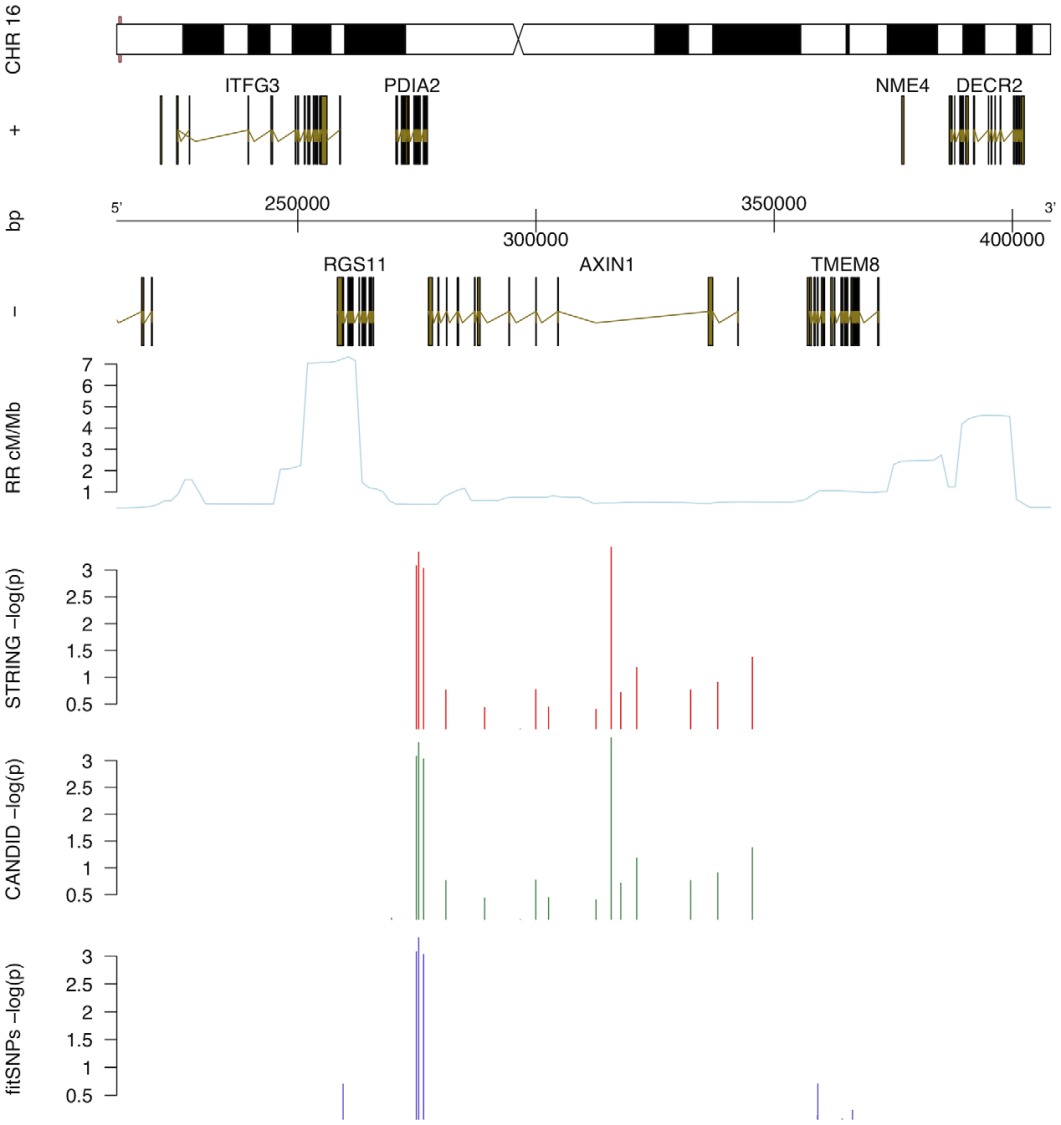
BAV Associated Haplotype Spanning PDIA2 and AXIN1. Data from three approaches and relevant genomic features extracted from the Ensembl 54 database are depicted. At the top of the plot is an ideogram depicting a location on chromosome 16; the small red box delimits a region between base pair 212416 and 407490, displayed immediately below the ideogram (track labeled “bp” which also indicates the 5′ to 3′ orientation of the plot). Annotated gene content is displayed on positive (denoted by “+”) and negative (denoted by “−”) strand. The four graphical data-panes indicate RR cM/Mb: relative recombination rate in centimorgans per megabase as derived from HapMap build 36. STRING −log(p), CANDID −log(p), and fitSNPs −log(p): −log_10_ uncorrected p-values observed in each of the three indicated schemes. All probes analyzed in the region by each respective schema are represented by a peak. The region between the two tallest peaks in the STRING and CANDID plots delineates the observed haplotype detailed in Figure 4.

**Figure 4:**
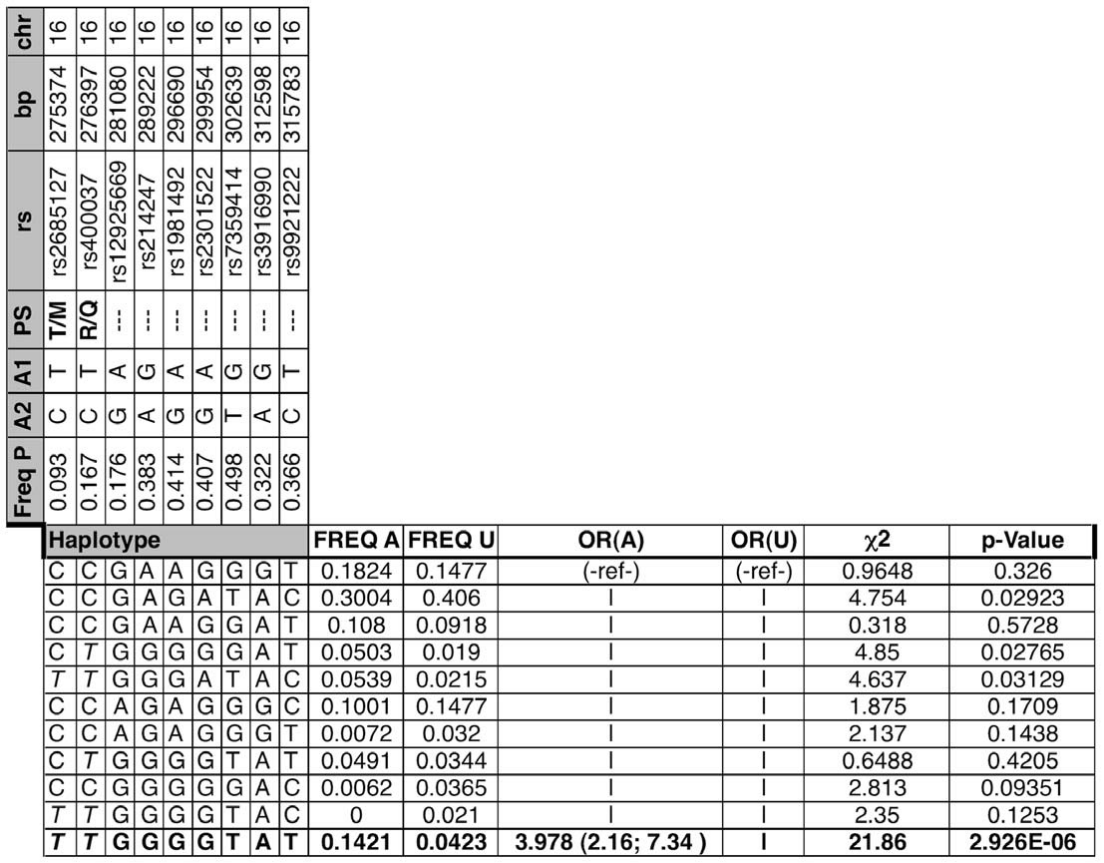
Haplotype Analysis for PDIA2 and AXIN1. “|” indicates haplotype grouped with one above it. Numbers in parenthesis represent 95% confidence interval. OR (A) and OR (U) abbreviate Odds Ratio Affected and Unaffected, respectively. Freq A, U, and P observed haplotype frequency Affected, Unaffected, and Population (control and experimental, this study); PS = Peptide Shift, italics bases within haplotype signify presence of peptide-shifting variant.

STRING, CANDID and FitSNPs identified a concentration of SNPs in chromosome 16p13.3 within a region that includes AXIN1 and PDIA2. AXIN1 was selected by CANDID and STRING because its expression in aorta from subjects with BAV compared with TAV was significantly different by limma (adjusted *p*-value of 3.79×10^−49^). Four SNPs selected by all three approaches within this region (Figure 2) showed associations with BAV that did not surpass correction for multiple testing (Figures 2 and 3). There was significant linkage disequilibrium observed in the locus suggesting a haplotype block structure. Eleven haplotypes derived from nine AXIN1-PDIA2 region SNPs (Figure 4) were identified in this region. The observed block structure in our case-control cohort recapitulates that observed by the relevant European HapMap data [29]. The TTGGGGTAT haplotype showed the strongest association with BAV and surpassed the Bonferroni correction for multiple testing (p-value 2.926×10^−6^, OR 3.978). Further narrowing or widening of this window reduced the association of the locus, strongly implying that variants within this regional block unit are associated to the BAV phenotype.

Haplotype analysis was unable to discriminate whether the variants in AXIN1 or PDIA2 were driving the association with BAV in this gene-rich region. Indeed, SNPs observed within PDIA2 are in high LD with SNPs in AXIN1 regulatory regions and vice-versa. However, the three SNPs with the strongest individual association with BAV are all non-synonymous polymorphisms of PDIA2: -T286M (rs2685127), -K185E (rs419949) and –Q388R (rs400037); of these, rs419949 lies outside the most strongly associated haplotype. These results suggest that the co-occurrence of two of these three PDIA2 protein-coding changes is associated with increased odds of BAV. However, as the bulk of the haplotype resides in AXIN1, a gene in a pathway relevant to heart valve formation,[30] we cannot exclude a role for a primary genetic variant located in either PDIA2, AXIN1, or both as contributing to the observed association with BAV. However, there are currently no additional known, non-synonymous coding SNPs located within this region beyond those represented in the collected genotypes.

### Endoglin (ENG) Haplotype Is Associated with BAV

**Figure 5:**
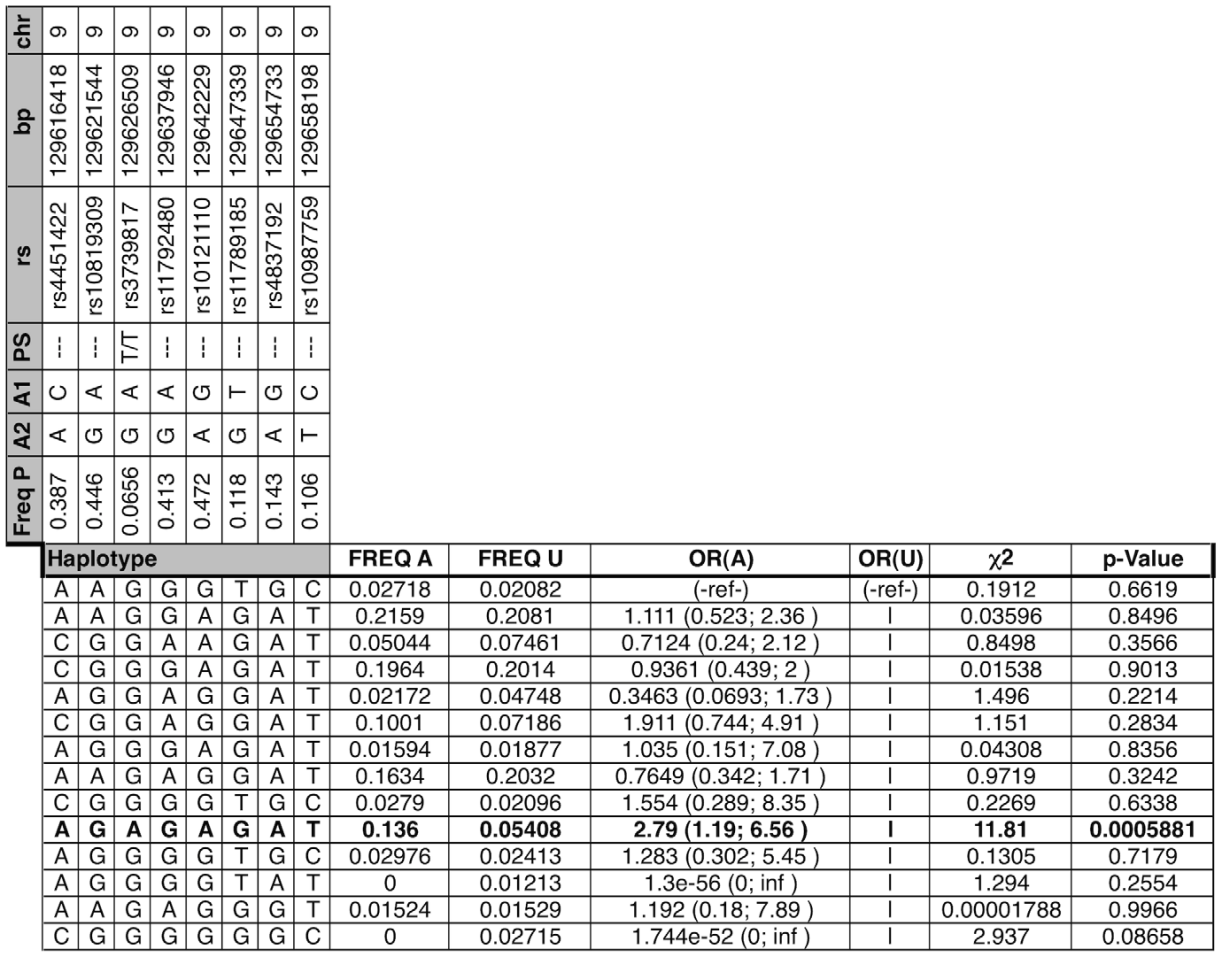
Haplotype Analysis for ENG. “|” indicates haplotype grouped with one above it. Numbers in parenthesis represent 95% confidence interval. OR (A) and OR (U) abbreviate Odds Ratio Affected and Unaffected, respectively. Freq A, U, and P observed haplotype frequency Affected, Unaffected, and Population (control and experimental, this study); PS = Peptide Shift (in this case a conservative instance), italics bases within haplotype signify presence of peptide-shifting variant.

SNPs within the gene endoglin (ENG) were prioritized for analysis by STRING, CANDID, and FitSNPs. ENG was initially selected for inclusion by RankProd analysis that found differential expression in aortic aneurismal tissue from patients with BAV compared with TAV (GDS2922). Haplotype analysis identified one block including a conservative coding region variant (ENG-T343T, rs3739817) associated with BAV (p-value 5.88×10^−04^, OR 2.79) (Figure 5). ENG-T343T (rs3739817) appears to be the critical SNP within the haplotype, as presence of the minor allele at this locus segregates with BAV across the region. Though the predicted amino acid change is synonymous (T/T), recent work suggests that even conservative alterations may yield functionally unique outcomes [31]. The possibility also exists for a novel, causative variation in high LD with this haplotype/SNP not present in our analysis; no known variants of this type are present within the region.

### Additional Loci

The BAT2/3 locus is located at chromosome 6, and two haplotypes in this region are each weakly associated with BAV (Table S2). However, detailed analysis of this haplotype fails to show a single, clear association with trait. Similarly, the remaining repeated-hit loci at MYLK, LEF1, CSF1R, REXO4, CD44, ZBTB16, and FBNL1, though containing associated SNPs, do not return a single associated haplotype that spans the region of interest, typically due to low probe counts, very large regions, or low frequency of the putative associated haplotypes within our population. While these loci may be significant in BAV, further study is required to fully clarify the location and identity of causative variant(s) that may be within these regions.

### Random Forests Analysis

**Figure 6:**
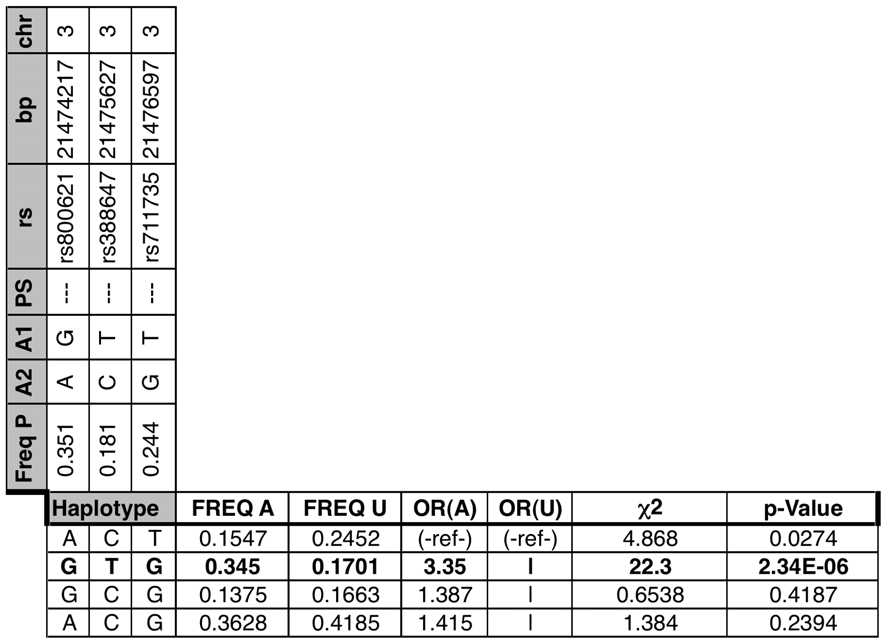
Haplotype Analysis for ZNF385D. “|” indicates haplotype grouped with one above it. Numbers in parenthesis represent 95% confidence interval. OR (A) and OR (U) abbreviate Odds Ratio Affected and Unaffected, respectively. Freq A, U, and P observed haplotype frequency Affected, Unaffected, and Population (control and experimental, this study); PS = Peptide Shift (in this case a conservative instance), italics bases within haplotype signify presence of peptide-shifting variant.

As a complementary strategy that is not dependent on existing knowledge we performed a Random Forests analysis to select SNPs from our genome-wide dataset with the greatest information content. Starting with 267,196 SNPs Random Forests analysis yielded an analysis group of 6,322 SNPs, approximately equal in dimension to that used by the supervised approaches. Fewer than five percent of the SNPs selected by Random Forests were also selected by either CANDID, STRING or fitSNPs (Table 1). Association analysis identified one SNP associated with BAV (rs388647, OR 4.562, *p* = 0.03201 following correction for multiple testing) located on chromosome 3 within the RefSeq transcript zinc finger protein 385D (ZNF385D; NM_024697.1). Low probe density in this region on the Illumina 370CNV array (there are only two other probes located within 5 kb of the associated SNP) prevents a more detailed haplotype analysis. However, the associated SNP and its two nearest neighbors form a stable haplotype that spans an exon (Figure 6).

### Comparison of the Four Gene/SNP Selection Strategies

We compared the top SNPs selected by the four gene/SNP selection strategies (Table S3). Each selection strategy picked a different top SNP. CANDID selected the SNP with the strongest BAV association (rs9930956).

## Discussion

These results demonstrate that mining existing published literature, expression, and genome scan data in a systematic manner can identify biologically plausible genes and pathways that have variants significantly associated with BAV, ultimately a disease of early development. Because we describe a single cohort, we consider the results to be hypothesis generating. The successful identification of candidate SNPs/genes by our prioritization analyses now provides motivation for the collection and testing of additional cohorts for independent analysis.

Molecular pathways that regulate the cellular components of the developing heart valve have been identified [32]. In particular, cells derived from the cardiac neural crest are critical cellular constituents of the developing aortic valve and the great vessels [33]; defects within cardiac neural crest development have been suggested as underlying BAV [34]. Mutations in NOTCH1, a signaling pathway component important for the cardiac valve formation [32], have been found to cause complex congenital heart defects including BAV [35] and have also been associated with BAV in the context of aneurysm [36]. We did not find that SNPs within NOTCH1 are associated with BAV in our cohort. However, we did identify BAV-associated genes that lie within networks that contribute to valve formation and include NOTCH1. Taken together with previous published findings [8], our results suggest that genetic derangement of pathways that contribute to valve formation underlie formation of a BAV.

A locus containing AXIN1 and PDIA2 was retained by all methods, and haplotype analysis revealed a strong association with BAV (Figure 3). AXIN1 (AXis INhibitor 1) is a critical member of the Wnt pathway, which is one signaling pathway that regulates both heart valve formation [32] and cardiac neural crest development [37]. In addition to the Wnt pathway, AXIN1 influences signaling by the transforming growth factor beta (TGFβ) family [38], which is also a key regulator in the cardiac neural crest [39]. When the individual AXIN1-PDIA2 SNPs were combined into a region-spanning locus, significant association with trait is strong and consistent with the overall independent findings (Figure 2 and Figure 3). The three most associated SNPs are in medium to high linkage disequilibrium with each other, and reconstructing haplotypes across this region functions to segregate affected from unaffected individuals. The presence of two Protein Disulfide Isomerase family A, member 2 (PDIA2) peptide modifying SNPs in the first two positions of this haplotype (Figure 3) provides a compelling potential functional mechanism. It is difficult to speculate the potential role for the PDIA2 coding SNPs in BAV, as the role of PDIA2 in heart valve formation is not known. It is worth noting that each of the haplotypes containing one of these two shifts is at least moderately significant relative to the trait. Further genotyping is required to determine the relative contribution of AXIN1 and PDIA2 variants to BAV.

Endoglin (ENG), as summarized in Figure 5, represents an instance in which a minor allele resulting in a conservative peptide shift is observed in the only haplotype associated with trait (Figure 5). Recently, there has been an appreciation that synonymous coding region polymorphisms can have a significant effect through alterations of translational efficiency and the secondary structure of the transcript [31]. In humans, ENG is expressed in heart valves and the aorta [40] and its expression is increased in endocardial cushion mesenchyme during valve formation [41]. ENG is required for differentiation of neural crest cells into the smooth muscle cells that populate the aorta [42], and knockout mice heterozygous for ENG deletion show a tendency towards aneurism formation [43]. The durable haplotype association observed at this locus is particularly interesting because defects in NOTCH signaling, which are associated with BAV [7], [8], have defective basement membrane around the early aorta and show down-regulation of ENG [44]. The identification of a haplotype of ENG, a gene known to be important in heart valve formation, being associated with BAV illustrates the potential for strategies centered on networks and systems biology to identify biologically important associations from large SNP datasets that would likely have otherwise been overlooked by traditional GWAS analysis.

SNP selection by Random Forest returned different markers than the two knowledge-based strategies. It must be noted that computational limitations ultimately affect how many forests could be grown for our analysis, even in the context of our relatively small population with several hundred thousand probes. The modified RandomJungle approach [45] appears to deal specifically with this limitation, and we anticipate investigating its utility in studies like those that we report here. We consider our identification of a SNP in chromosome 3 associated with BAV to be hypothesis generating pending replication in independent cohorts because the SNP occurs in a relatively low-probe-density region and the marginal significance of the null-model haplotype weakens the likelihood of overall locus significance. While further analysis is needed to establish the durability of this isolated observation, the presence of a strong haplotype across a coding region implies a potential functional outcome, the biological impact of which remains uncertain.

Probe prioritization in our case offered *at least* an order of magnitude of multiple-testing relief. In the case of the CANDID probe collection (which employs the largest number of probes of any set used in this study) the probe prioritization process reduced the fraction of the genome analyzed to 0.19% of the total. This directed specificity is not reflected in current, overly strict correction methodology. Further complications emerge in the apparent genetic heterogeneity underlying the singular phenotype of BAV. While several apparently causative defects have been identified in isolated families, the broader spectrum of BAV has been difficult to characterize genetically [9], [4], [5]. We hypothesize that any cohort of BAV individuals is likely to be a mixed representation of various possible BAV-causing developmental defects, further eroding the capability of small cohorts to describe the condition fully at the genetic level. We describe a method for prioritization of specific SNPs that both directly addressed these potential issues and successfully identified biologically plausible genomic loci associated with BAV. The identification of genes with known biological roles in heart valve formation (AXIN1 and ENG) and additionally within pathways and regulatory networks previously associated with BAV in family-based studies demonstrates the power of association testing that incorporates biological knowledge. The findings presented here highlight the need for collection and analysis of a large BAV cohort powered for a fully unbiased analysis and showcases the power that gene-based and network-based analyses can supply for analyzing complex but heritable genetic systems.

## Materials and Methods

### Cohort

The Tufts Medical Center/Tufts University Institutional Review Board approved all studies and study procedures followed signed participant informed consent in accordance with the principles expressed in the Declaration of Helsinki. Probands were identified by the discovery of a bicuspid aortic valve at the time of aortic valve replacement or defined by echocardiography, agreed upon by two separate readers. The average age of BAV Genetics Study participants at the time of study entry was 53 years (range 18 to 85), and the majority of subjects are men (54 of 66). Among the 66 subjects with a BAV, six also had coarctation of the aorta (all repaired) and 19 were found to have an aneurysm of the ascending aorta (17 repaired). Echocardiography demonstrated normal valve function of the BAV in 18 subjects. Moderate to severe aortic valve stenosis was detected in 38 subjects; three subjects had isolated severe aortic valve regurgitation. Genomic DNA was genotyped on the Illumina 370CNV array by deCode (Reykjavik, Iceland). Genotypes for 823 control individuals (average age 43, range 30 to 88) were obtained from Illumina iControlDB [23]. These individuals are genotyped on the same platform as our experimental study was with the exception of copy number probes present on the 370CNV array variant that were not included in the control population study; all non-overlapping probes were set to missing for the purposes of this study. When these individuals were added to the handful of familial cases and controls (a total of five cases (∼7%) exhibit family history of BAV) already in our population, 830 control individuals were available for use in the analysis. Using the software package *Structure* and the standard methodology described by Pritchard *et al.* [24], we employed 25,000 independent simulations to model the number of potential sub-populations (*K*) present in our case/control cohort. The smallest value of *K* that appeared to describe the cohort was 3. Values of *K* from 0 to 8 were analyzed for their consistency. These analyses returned no evidence of significant allele frequency divergence at any value of *K* within the control and experimental groups when considered singly or as a whole.

### Power to Detect Associations

Using the *Genetic Power Calculator* for discrete traits [46], we determined that, with our cohort dimensions and a 1% population prevalence of BAV, power to detect at a significant association in our cohort would reach 80% at observed odds ratios greater than 2. Odds ratios of 4 or greater (as observed in the AXIN1 haplotype, Figure 4) reach similar power with 53 individuals. By comparison, the various BAV sub-phenotypes present in our cohort each represent fewer than 18 individuals, far fewer than are required to reach any reasonable power threshold; these sub-phenotypes were therefore excluded from further analyses.

### DNA

Collection of DNA from subjects with a BAV, and some of their family members, began February 2006. Cases were identified at the time of aortic valve replacement by direct surgical observation or from an echocardiogram performed for evaluation of the aortic valve. Blood is collected in PaxGene DNA tubes (Qiagen) and the DNA is purified using a dedicated purification kit. The concentration of DNA in each sample is measured using the PicoGreen assay (Invitrogen).

### Gene, Interaction Network, and SNP Selection

*1) Generation of the initial phenotype descriptor*. Using Cytoscape's Agilent Literature Search plug-in [47], we employed a list of twenty broadly topical MeSH keywords to describe each of the major categories and outcomes of BAV and BAV-associated syndromes (the complete list is available in Supplemental Methods). Additionally, we added various gene-specific descriptors, such as “NOTCH,” to ensure full coverage of previously described BAV genetic profiles. We also included information garnered from microsatellite analysis of BAV individuals, which had identified several discrete regions of the genome thought to be involved in the development of BAV [27]. These core terms produced a basic protein interaction network consisting of 124 proteins (and genomic locations). *2) Addition of prioritized genes*: We analyzed GDS2922 and GSE9106 separately using limma [48] and RankProd [49] applying default settings within the statistical analysis program R [50]. 3) *STRING and CANDID analysis*: To integrate and extend the RankProd and limma generated gene lists with the initial phenotype descriptor network we employed STRING and CANDID.

#### STRING and CANDID network integration

The intention of using dual approaches being to capture both broadly modified genes between classes as well as those genes that vary within classes while maintaining an overall differential expression level between the two groups. With regard to STRING, networks were formed with a maximum of 4 additional inter-member nodes, a “medium” confidence score of 0.4, no more than 50 interactors shown, an edge scaling of 80%, and all Active Prediction Methods selected. CANDID allows for more nuanced subscoring of various components. All components were weighted equally, with the exception of “Conservation,” which was given the lesser weight of 2 relative to the rest of the entries. Tissue codes 44 (heart) and 53 (cardiac myocytes) were employed.

In all cases, output of proteins and their interactors were converted into genomic regions of origin by employing the UCSC Genome Table Browser, employing Ensembl Gene codes (ENSG) to capture various potential transcripts that may arise from a single “gene.” Additionally, a buffer region of 5 kb was added to the beginning and end of each expression region to allow for upstream or downstream control elements potentially present. These regions in hand, the intersection of CNV370 probes falling within the various regions were calculated. The number of SNPs corresponding to each class of prioritization with relative overlap between classes is available in Table 1.

### Random Forests

Carried out using the R package randomForests [51] with the following non-default settings: 10,000 trees, 5,000 iterations, and importance = TRUE. Input data comprised all SNPs with a non-zero variance. Total input SNPs numbered 267,196 yielding 6322 “important” SNPs for further analysis.

#### Association and locus analysis

These analyses were carried out using plink v1.06 [52]; baseline settings were employed for inclusion of probes and quality control. (genotyping rate≥80%, pruning of probes based on missingness (GENO≥1) and low frequency (MAF≤0) as well as the removal of heterozygous haploid genotypes (4545 found and removed from analysis in this dataset). Total genotyping rate across all individuals (66 cases, 830 controls) was 0.98, and after all pruning the initial probe number (from which all described priority and GWAS probes were drawn) was 311,399. Primary analysis of all priority sets (as well as GWAS) was conducted using the same set of covariates and phenotype definitions for each set. Full plink settings for each run were “—logistic –adjust –qq-plot –sex” with the requisite additional commands for selecting input and output files. Logistic analysis is indicated for the discrete disease trait (BAV/no-BAV) and the covariate adjustment is included for gender and, ultimately, for returned p-values relative to number of tests performed. Lack of deep phenotype information for control population limits the ability to create more extensive covariate controls in this population.

## Supporting Information

Table S1 Frequency of major gene ontology classes present in the combined prioritized gene lists returned by CANDID and STRING. Frequency counts and fractional percentage relative to total number of observations. Pathway members lacking more specific annotations return the generic “biological_process” heading.
(0.04 MB PDF)

Table S2 Haplotype Analysis for BAT2/3. “|” indicates haplotype grouped with one above it. Numbers in parenthesis represent 95% confidence interval. OR (A), OR (U) Odds Ratio Affected and Unaffected, respectively. Freq A, U, and P observed haplotype frequency Affected, Unaffected, and Population (control and experimental, this study); PS = Peptide Shift (in this case a conservative instance), italics bases within haplotype signify presence of peptide-shifting variant.
(0.04 MB PDF)

Table S3 Lowest observed significant corrected p-Values by prioritization category. Lowest corrected p-Value observed at SNP is bolded; NP = Not Present in group.
(0.04 MB PDF)
