# Supplementary material for: Application of Gene Network Analysis Techniques Identifies AXIN1/PDIA2 and Endoglin Haplotypes Associated with Bicuspid Aortic Valve
Source: PLoS One. 2010 Jan 21;5(1):e8830. doi: 10.1371/journal.pone.0008830 (PMC2809109; doi:10.1371/journal.pone.0008830)
Supplement: Table S1 — Frequency of major gene ontology classes present in the combined prioritized gene lists returned by CANDID and STRING. Frequency counts and fractional percentage relative to total number of observations. Pathway members lacking more specific annotations return the generic “biological_process” heading. (0.04 MB PDF) [file pone.0008830.s001.pdf]

GO Class ID	Definitions	Counts	Fraction
GO:0008150	biological_process	357	35.45%
GO:0007275	development	74	7.35%
GO:0008152	metabolism	73	7.25%
GO:0007154	cell communication	50	4.97%
GO:0007165	signal transduction	44	4.37%
GO:0030154	cell differentiation	43	4.27%
GO:0019538	protein metabolism	35	3.48%
GO:0009653	morphogenesis	32	3.18%
GO:0016043	cell organization and biogenesis	31	3.08%
GO:0006810	transport	22	2.18%
GO:0009790	embryonic development	21	2.09%
GO:0008219	cell death	18	1.79%
GO:0016265	death	18	1.79%
GO:0006139	nucleobase, nucleoside, nucleotide and nucleic acid metabolism	16	1.59%
GO:0009058	biosynthesis	15	1.49%
GO:0006464	protein modification	13	1.29%
GO:0006950	response to stress	13	1.29%
GO:0008283	cell proliferation	13	1.29%
GO:0009605	response to external stimulus	13	1.29%
GO:0006350	transcription	11	1.09%
GO:0000003	reproduction	9	0.89%
GO:0006519	amino acid and derivative metabolism	7	0.70%
GO:0006412	protein biosynthesis	6	0.60%
GO:0006629	lipid metabolism	6	0.60%
GO:0015031	protein transport	6	0.60%
GO:0019725	cell homeostasis	6	0.60%
GO:0006996	organelle organization and biogenesis	5	0.50%
GO:0007267	cell-cell signaling	5	0.50%
GO:0009056	catabolism	5	0.50%
GO:0009607	response to biotic stimulus	5	0.50%
GO:0016032	viral life cycle	5	0.50%
GO:0005975	carbohydrate metabolism	4	0.40%
GO:0006811	ion transport	4	0.40%
GO:0007610	behavior	4	0.40%
GO:0007005	mitochondrion organization and biogenesis	3	0.30%
GO:0009628	response to abiotic stimulus	3	0.30%
GO:0040007	growth	3	0.30%
GO:0006259	DNA metabolism	2	0.20%
GO:0007049	cell cycle	2	0.20%
GO:0006091	generation of precursor metabolites and energy	1	0.10%
GO:0007028	cytoplasm organization and biogenesis	1	0.10%
GO:0008037	cell recognition	1	0.10%
GO:0009719	response to endogenous stimulus	1	0.10%
GO:0016049	cell growth	1	0.10%
