# Supplementary material for: Application of Gene Network Analysis Techniques Identifies AXIN1/PDIA2 and Endoglin Haplotypes Associated with Bicuspid Aortic Valve
Source: PLoS One. 2010 Jan 21;5(1):e8830. doi: 10.1371/journal.pone.0008830 (PMC2809109; doi:10.1371/journal.pone.0008830)
Supplement: Table S2 — Haplotype Analysis for BAT2/3. “|” indicates haplotype grouped with one above it. Numbers in parenthesis represent 95% confidence interval. OR (A), OR (U) Odds Ratio Affected and Unaffected, respectively. Freq A, U, and P observed haplotype frequency Affected, Unaffected, and Population (control and experimental, this study); PS = Peptide Shift (in this case a conservative instance), italics bases within haplotype signify presence of peptide-shifting variant. (0.04 MB PDF) [file pone.0008830.s002.pdf]

chr	bp	rs	PS	A1	A2	Freq P
6	31701455	rs2260000	---	C	T	0.448
6	31705732	rs2242660	---	T	C	0.345
6	31709822	rs3115663	---	G	A	0.141
6	31710946	rs1046089	R/H	A	G	0.278
6	31711570	rs2261033	---	T	C	0.457
6	31711749	rs9267522	---	G	A	0.141
6	31712023	rs3132453	---	A	C	0.0356

Haplotype							FREQ A	FREQ U	OR(A)	OR(U)	$\chi 2$	p-Value
C	C	A	G	C	A	C	0.2973	0.3886	(-ref-)	(-ref-)	3.791	0.05154
T	C	A	G	T	A	C	0.1535	0.1714	1.079 (0.603; 1.93)	I	0.2434	0.6218
**T**	**T**	**G**	***A***	**T**	**G**	**C**	**0.2261**	**0.1344**	**2.192 (1.28; 3.76)**	**I**	**7.496**	**0.006184**
T	T	A	*A*	C	A	C	0.09872	0.09219	1.285 (0.63; 2.62)	I	0.05462	0.8152
**T**	**C**	**A**	**G**	**T**	**A**	**A**	**0.06957**	**0.03344**	**2.555 (1.11; 5.86)**	**I**	**4.064**	**0.0438**
T	T	A	G	C	A	C	0.02797	0.07116	0.5033 (0.157; 1.61)	I	3.15	0.07595
C	C	A	G	T	A	C	0.07771	0.06547	1.526 (0.683; 3.41)	I	0.2605	0.6098
T	T	A	*A*	T	A	C	0.0491	0.04339	1.492 (0.564; 3.95)	I	0.08396	0.772
