# Supplementary material for: Application of Gene Network Analysis Techniques Identifies AXIN1/PDIA2 and Endoglin Haplotypes Associated with Bicuspid Aortic Valve
Source: PLoS One. 2010 Jan 21;5(1):e8830. doi: 10.1371/journal.pone.0008830 (PMC2809109; doi:10.1371/journal.pone.0008830)
Supplement: Table S3 — Lowest observed significant corrected p-Values by prioritization category. Lowest corrected p-Value observed at SNP is bolded; NP = Not Present in group. (0.04 MB PDF) [file pone.0008830.s003.pdf]

SNP	CHR	BP	UNADJ	fitSNP BONF	CANDID BONF	STRING BONF	RF BONF	FDR
rs388647	3	21475627	5.06E-06	NP	NP	NP	**0.03201**	0.02099
rs942379	6	25957598	4.02E-06	**0.02937**	NP	NP	NP	0.01468
rs2596501	6	31429189	1.68E-06	NP	NP	**0.01475**	NP	0.0147
rs9930956	16	2025295	5.50E-08	NP	**0.0006906**	NP	NP	0.0006906
rs2290902	16	87310176	3.27E-06	**0.02391**	NP	NP	NP	0.01468

Supplemental Table 3: Lowest observed significant corrected p-Values by prioritization category. Lowest corrected p-Value observed at SNP is bolded; NP= Not Present in group.
